# Developing and Delivering a Regional Teaching Programme in Liaison Psychiatry: A Quality Improvement Project

**DOI:** 10.1192/bjo.2024.447

**Published:** 2024-08-01

**Authors:** Sally Tulip, Cara Katona

**Affiliations:** Camden and Islington NHS Foundation Trust, London, United Kingdom

## Abstract

**Aims:**

Several sites across the North London Mental Health Partnership (NLMHP) do not have a liaison-specific rolling teaching programme. Best practice standards set by the RCPsych Psychiatric Liaison Accreditation Network (PLAN) are therefore not being met.

The aims of this quality improvement project (QIP) were to: (1) ascertain the perceived need for liaison-specific teaching across NLMHP sites; (2) develop and deliver a teaching programme; and (3) assess attendance, clinician satisfaction and confidence before and after teaching sessions.

**Methods:**

A pre-programme questionnaire on Microsoft Forms was sent to team members across NLMHP sites to assess whether respondents were receiving liaison-specific teaching, the perceived utility of the programme, and suggestions for development.

A cross-site monthly teaching programme was developed. Sessions were presented by liaison clinicians from a list of liaison-specific topics via Microsoft Teams.

A post-session questionnaire was sent to establish session satisfaction, confidence pre- and post-session, and further comments. Mean satisfaction scores were calculated. Percentage change in confidence score was calculated for each session and overall.

Themes were identified from the qualitative data and suggestions implemented.

**Results:**

Of the 11 professionals who responded to the pre-programme questionnaire, 50% were not receiving any liaison-specific teaching. Respondents agreed the programme would be helpful in improving their knowledge and clinical practice (mean score = 4.9/5).

Attendance for the sessions ranged from 15–27 professionals (mean = 22). A range of 2–10 professionals completed each post-programme questionnaire (mean = 6.3; total responses = 25). Mean satisfaction for each session ranged from 4.3–5/5 (overall mean = 4.7/5). Percentage increase in confidence scores ranged from 4.6–48% (mean = 24%).

Feedback-driven changes made to improve the programme included: making session recordings available; sending reminder emails; creating an online platform and making session feedback available to presenters.

Respondents considered the sessions interesting and informative, that topics provoked good discussion, and that the 'bite-sized' training allowed attendance without interfering with clinical work.

**Conclusion:**

This QIP highlighted the need for a liaison-specific teaching programme across NLMHP. Participants agreed that this would improve their knowledge and practice. The programme was reasonably well-attended across sites. Respondents reported improved confidence and felt the sessions were relevant to their clinical practice.

Limitations included the low and variable questionnaire response rate and limited data on the new programme's utility.

The next stages of the project include wider delivery, involvement of patients and carers, and of specialists in related psychiatric and medical fields.

